# Remarkable Complication of Diseases

**Published:** 1850-04

**Authors:** Claiborne Pirtle

**Affiliations:** Louisville, Ky.


Art. V.—*Remarkable Complication of Diseases.* By Claiborne Pirtle, M. D., of Louisville, Ky.

On the 30th January, 1850, I was called to see William Poutch, a lad aged fourteen years.

I learned from his family that he came home the day before complaining of very great pain in his knees and elbows, extending up the arms, and it was with great difficulty he could walk or use his arms. I found him on my first visit pretty much as I was told he had been the day before, when he came home from his work in the foundery. Upon examination I found great tenderness and soreness of the knees and elbows, extending up the arms. There was neither swelling, heat, nor redness of the parts complained of, but they were painful when moved. The tongue was coated with a yellowish fur; the pulse excited but little, if any; the face was somewhat flushed and covered with a gentle perspiration; bowels rather inactive; no headache. I ordered the following prescription: ℞ Hydr. submur. grs. viii, sulph. quinine grs. iv, pulv. opi. gr. ss. mix, make iv pills; one pill to be given every two hours.

January 31. The medicine had not moved the bowels; great complaint of pain in the abdomen, but there is no disposition to evacuate the bowels; knees, arms, and wrists painful; has vomited several times this morning; great thirst; pulse about the natural frequency but feeble; no fever; tenderness and pain over the abdomen upon pressure, particularly over each iliac region. Ordered an enema of castor oil and spirits turpentine to be given in thin starch water, and thus succeeded in producing three motions upon the bowels, of a consistent bilious character.

Evening.—Pain still continues in the bowels, with repeated vomitings. Ordered, to be given through the night, a pill of camphor and opii, to be repeated frequently until the bowels or pain in the abdomen should be relieved. Sinapisms of mustard were ordered for the abdomen.

February 1st.—Found the patient easier than the day before; had slept some and did not vomit. While examining him this morning, I found his ears livid or of a purplish hue, and the skin evidently in a state of deep congestion. He told me that there was a breaking out upon the tops of his thighs, and, upon examination, I found the nates and gluteal region covered with a great many purple spots, some of them, perhaps all, some little elevated above the surface of the surrounding skin. I ascertained the existence of similar purple spots upon various other parts, and especially upon each elbow and ankle. On examining the mouth I discovered a sprinkling of these spots upon the epithelium of the lips and gums and palate. There is no fever, but the thirst continues. Ordered sulph. quinine ℈i, carb. ammonia ʒi, simple syrup ℥ij, paregoric ℥j, mix; dose, one teaspoonful every two hours. On my visit in the evening I found that the gastric symptoms had come on again, and that everything taken into the stomach had been vomited up. Thirst very great. Directed ice to be swallowed, and ordered hot cloths, wrung out of heated spirits, to be applied to the abdomen; discontinued all medicine internally.

February 2.—The patient had a bad night; slept but little; vomiting continues; considerable tympanitis and thirst; pulse frequent and feeble. Ordered an enema, but did not succeed in opening the bowels. Gave brandy and water; directed warm flannel to the bowels; the stomach has constantly rejected all nourishment.

February 3.—All the symptoms continue as on yesterday; bowels not moved; tympanitis considerable; everything taken into the stomach is thrown up. Ordered eight leeches to the epigastrium, and sinapisms and hot fomentations over the bowels. Directed chicken water as diet.

February 4.—Found the patient worse; he was throwing from the stomach a fetid, colored fluid, indicating a reversed action of the peristaltic motion of the bowels. There was a peculiarly anxious expression of countenance. An enema of castor oil and turpentine was given in thin starch water, and a discharge of hardened feces was obtained. Prescribed creosote mixture. Gangrenopsis showed itself in full force about this time.

February 5.—More quiet this morning; slept well last night, but manifested a marked typhoid state of the nervous system. Fluid magnesia, with as much camphor as it would hold in solution, was ordered to be given frequently through the day. In the evening found the sufferer sleeping soundly; has not vomited since some time in the night; tympanitis has almost disappeared, but there is fullness in the epigastric region; knees not so painful; no particular suffering complained of at any point to-day.

February 6.—On my visit this morning I found him very restless again. Pulse excited and some fever; commenced vomiting again this morning; left off the brandy; gave him, as before, ice freely. Saw him in the afternoon, with Dr. Bell; all his previous symptoms aggravated; vomiting the contents of the bowels; gangrenous condition of the mouth; fetid breath. Ordered charcoal and yeast to be given freely, and also Catawba wine, by the ounce, frequently repeated. A combination of castor oil and turpentine was given by the mouth, and injections of the same were directed.

February 7.—No change in the aggravated symptoms; nothing was retained on the stomach; great tension of that organ; the mind was rational; had slept some during the night; thirst great; expression sunken and anxious; as the injection had not been given, ordered it this morning; succeeded in opening the bowels; gave wine, but he died at three o'clock, P. M.

---

*Remarks.*—Dr. Pirtle has detailed, with minuteness, the principal features of the remarkable complication of disease in this case. In a long experience we never saw anything like it. The little victim was one of the most remarkable boys we have ever known. He was very industrious; free from everything like vice, temperate in all things, and we found it impossible to trace his diseases to violations of the laws of health, either in his trade, his mode of life, or the location of his dwelling. His parents have the appearance of being very healthy.

In these circumstances it is difficult to account for the complexity of diseased actions manifested in his case—purpura, gangrenopsis and typhoid fever were well marked in their presence.

The case is a curious one in pathology, and, as such, is submitted to the profession. A portion of its described features make it resemble some of Andral's cases of gastro-enteritis, but no one could have mistaken the case at the bedside.—*Ed. Western Journal of Medicine and Surgery.*

